# Reduced RCE1 expression predicts poor prognosis of colorectal carcinoma

**DOI:** 10.1186/s12885-017-3393-3

**Published:** 2017-06-14

**Authors:** Boyun Shi, Xinke Zhou, Lu He, Min Liang, Yuanwei Luo, Peng Jiang

**Affiliations:** 1 Department of Oncology, The Fifth Affiliated Hospital, Guangzhou Medical University, 621 Harbor Road, Guangzhou, Guangdong 510700 China; 2 Department of Radiotherapy, Affiliated Tumour Hospital, Guangzhou Medical University, Guangzhou, Guangdong China

**Keywords:** Colorectal carcinoma (CRC), RCE1, MAPK, Invasion, Prognosis

## Abstract

**Background:**

As an end-proteolytic enzyme that cleaves the last three residues of proteins with a terminal CAAX, Ras-converting enzyme 1 (RCE1) has an essential role in multiple signaling pathways and take part in the process of differentiation, proliferation and carcinogenesis. The aim of the study is to investigate expression pattern of RCE1 and its prognosis in colorectal carcinoma (CRC).

**Methods:**

The expression of RCE1 and phospho-MAPK family members was confirmed by immunohistochemical staining of CRC tissues. miR-RCE1 lentiviral vectors were transduced into HCT116 and SW489 cells. Reverse transcription PCR (RT-PCR) and western blot were conducted to measure the transfection efficiency. Transwell assays were used to detect the invasiveness of CRC cells.

**Results:**

In the present study, we assessed RCE1 expression in 244 CRC specimens and matching adjacent, non-tumorous tissues by immunohistochemistry (IHC). Compared with the matched adjacent non-tumor tissue samples, the RCE1 reduced in the tumor tissue samples (*p* < 0.001). RCE1 expression was significantly decreased in 106 of 244 (43.4%) CRC cases. In univariate and multivariate analyses, Decreasing expression of RCE1 independently predicts poor prognosis for patients in both overall survival and disease-free survival. Further study indicated that RCE1 influenced tumor invasion through the p38 pathway. Knockdown of RCE1 reduced phosphorylation and significantly increased the invasive capacity of CRC cells.

**Conclusion:**

Taken together, the outcomes of this study indicate that RCE1 acts as a tumor suppressor in CRC, as its reduced expression may increase CRC cell invasion and independently predict an unsatisfactory prognosis in CRC patients.

**Electronic supplementary material:**

The online version of this article (doi:10.1186/s12885-017-3393-3) contains supplementary material, which is available to authorized users.

## Background

Colorectal cancer (CRC) is one of the most common malignancies worldwide [1]. Despite great advances in medical management, the prognosis of patients suffered advanced disease remain poor [2, 3]. It was previously reported that the 5-year survival rate for patients with advanced stage CRC was less than 11% [4]. Thus, identifying new prognostic biomarkers would help both to estimate risk and to develop treatment plans.

As an integral membrane protease of the endoplasmic reticulum, Ras-converting enzyme 1 (RCE1) is classified as a member of the metalloproteinase family [5]. The main function of RCE1 is to process the CAAX motifs on the C-termini of some CAAX proteins, such as the Ras superfamily of small GTPases, the the γ-subunit of heterotrimeric GTPases, nuclear lamins, several protein kinases and phosphatases [6, 7]. These proteins are involved in mulltiple processes, including differentiation and proliferation. Acculumulating evidence suggests that RCE1 plays an important role in cancer development [8]. Moreover, it has been reported that RCE1 was required for membrane localization and activation of Ras [9, 10]. It is well known that Ras mutations and abnormal activation contribute to the development of several types of cancer, including CRC [11]. RCE1 might participate in Ras activation; However, the role of RCE1 genes in colorectal carcinoma has not been explored. Thus, we detected RCE1 expression and evaluated its prognostic significance in 244 CRC samples. Furthermore, we also investigated the molecular mechanisms in which RCE1 might be involved.

## Methods

### Patients and specimens

We collected tissues from 244 patients afflicted with colorectal carcinoma who were treated at the Affiliated Tumor Hospital of Guangzhou Medical University (Guangzhou, China) from january 2009 to December 2011. This study was approved by the Institutional Review Board and Human Ethics Committee at Affiliated Tumor Hospital of Guangzhou Medical University, Consent to use paraffin embeded colorectal tissue specimens for the intended research was obtained from all patients or their families. All of the patients had undergone curative resection, and the final pathological diagnosis was adenocarcinoma. In addition, those patients were followed up after surgery untill 10 November 2016. A total of 244 colorectal carcinoma samples, including matched adjacent non-tumorous disease, were used for immunohistochemistry (IHC) analysis. The detailed clinicopathological parameters are listed in Table 1. Overall survival (OS) defined as the time of surgery to the date of death or the latest follow-up. Disease-free survival (DFS) was defined as the time from the surgery to the date of local failure/distant metastasisi or the date of death or latest follow-up. The local failure-free survival (LFFS) and metastasis-free survival (MFS) were defined as the date of local failure or distant metastasis, respectively, or the date of death or when censored at the latest follow-up.

**Table 1:** Correlation of RCE1 and p-p38 protein expression with clinicopathological parameters

Characteristics	n	RCE1		*P-*value	P-p38		*P*-value
		Low	High		Low	High	
Gender							
Female	96	36 (37.5%)	60 (62.5%)	0.113	43 (44.8%)	53 (55.2%)	0.190
Male	148	72 (48.6%)	76 (51.4%)		80 (54.1%)	68 (45.9%)	
Age (y)							
> 60	105	40 (38.1%)	65 (61.9%)	0.118	51 (48.6%)	54 (51.4%)	0.698
≤ 60	139	68 (48.9%)	71 (51.1%)		72 (51.8%)	67 (48.2%)	
CEA (ng/ml)							
> 5	116	63 (54.3%)	53 (45.7%)	**0.003**	67 (58.0%)	49 (42.0%)	**0.030**
≤ 5	128	45 (35.2%)	83 (64.8%)		56 (43.8%)	72 (56.2%)	
CA19–9 (U/ml)							
> 37	59	30 (50.8%)	29 (49.2%)	0.107	30 (50.8%)	29 (49.2%)	**0.021**
≤ 37	185	78 (42.2%)	107 (57.8%)		93 (50.3%)	92 (49.7%)	
Location							
colon	121	56 (46.3%)	65 (53.7%)	0.797	64 (52.9%)	57 (47.1%)	1.000
Rectum	123	51 (41.5%)	71 (58.5%)		59 (48.0%)	64 (52.0%)	
Depth of invasion							
T1/T2	58	15 (25.9%)	43 (74.1%)	**0.001**	18 (31.0%)	40 (69.0%)	**0.001**
T3/T4	186	93 (48.7%)	93 (51.3%)		105 (56.5%)	81 (43.5%)	
Histological grade							
I/II	214	95 (44.4%)	119 (55.6%)	1.000	108 (50.5%)	106(49.5%)	1.000
III	30	13 (43.3%)	17 (56.7%)		15 (50%)	15(50%)	
Node stage							
N0	156	58 (37.2%)	98 (62.8%)	**0.000**	79 (50.6%)	77 (49.4%)	0.790
N1	59	26 (44.1%)	33 (55.9%)		28 (47.5%)	31 (52.5%)	
N2	29	24 (82.8%)	5 (17.2%)		16 (55.2%)	13 (44.8%)	
TNM stage							
I	48	11 (22.9%)	37 (77.1%)	**0.001**	17 (35.4%)	37 (64.6%)	**0.04**
II	108	48 (44.4%)	60 (55.6%)		62 (57.4%)	46 (42.6%)	
III	88	49 (56.7%)	39 (43.3%)		44 (50%)	44 (50%)	

Bold values **(** *p* < 0.05) indicate statistical significance

### Immunohistochemical analysis

The colorectal carcinoma specimens fixed by formalin and embedded by paraffin were sliced into 4-μm sections. After incubated at 60 °C for 2 h, the specimens were deparaffinized in xylene and then rehydrated with graded alcohols. To block endogenous peroxidase, We treated the tissue slides with 3% hydrogen peroxide in methanol for 15 min. And antigen retrieved was performed in sodium citrate buffer (pH 6.0) using a microwave oven. Before incubated with primary antibodies overnight at 4 °C, 1 h of preincubation in goat serum was performed to block non-specific staining. The primary antibodies used for the IHC assays were as follows: rabbit antibody against RCE1 (Santa Cruz Biotechnology, Dallas, TX, USA) and rabbit antibodies against p-p38, p-ERK1/2 and p-JNK (Cell Signaling Technology, Danvers, USA). According to the manufacturer’s instructions (DAKO, Glostrup, Denmark), The tissue slides were treated with a non-biotin horseradish peroxidase detection system. Two different pathologists who were specialized in colorectal cancer and blinded to the tissue type and clinical data evaluated the RCE1 IHC results. The intensity and extent of staining were taken into consideration. The staining intensities was rated from 0 to 3, and the staining extent was rated from 0% to 100%. The final score of each staining was obtained by multiplying the two scores [12, 13]. RCE1 expression was classified as positive if the score was higher than 1.5; if the score was 1.5 or lower, the case was classified as negative expression. p-p38, p-JNK and p-ERK1/2 were considered to be positively if the scores was higher than 0.5.

### Cell culture and Lentiviral infection

The colorectal cancer cell lines SW480(ATCC Cat. Number:CCL-247™) and HCT116(ATCC Cat. Number:CCL-1642™) were purchased from the American Type Culture Colletion (ATCC, Manassas, USA). SW480 and HCT116 were cultured in RPMI 1640 (Thermo Scientific, Waltham, MA) at 37 °C in a humidified atmosphere that contained 5% CO_2_. Media was supplemented with 10% fetal bovine serum, 50 mg/mL streptomycin, and 50 U/mL penicillin. The lentivirus targeting RCE1 was obtained from GenePharma (Shanghai, China), and the lentiviral infection was performed following the manufacturer’s protocol.

### Real-time PCR (RT-PCR)

RT-PCR was performed as previously described [14]. Briefly, total RNA was treated with DNase I (TaKaRa), and 2-μg aliquots were used for cDNA synthesis using random hexamers with Superscript III (Invitrogen). PCR amplification was performed by using the cDNA templates. The RCE1 primers were as follows: forward primer, 5′-CAGCTCTCTATGGATTGCCCT-3′; reverse primer, 5′-CGGGGCGATCACTTGGTTC-3′.

### Western blot

To prepare the cell lysates, 2–3 × 10^6^ cells were washed with ice-cold PBS and lysed using 1% Triton-X100 in PBS-T (0.1% Tween20 in PBS) in the presence of a 1× protease inhibitor. After incubation on ice for 10 to 15 min, whole cell lysates were centrifuged at 13,000 RPM at 4 °C for 20 min. The Pierce BCA Protein Assay Kit (Thermo scientific) was used for detect the concentration of total protein. Equal amounts of protein (20–25 μg) from each sample were mixed with loading buffer and then heated at 95 °C for 5 min. The samples were separated by electrophoresis on a 10% SDS-polyacrylamide gel at 100 V for 1 h, and they were then electrotransferred onto Hybond ECL nitrocellulose membrane (GE Healthcare) at 40 V for 1.5 hours. In order to prevent non-specific staining, the membranes were blocked with 5% skimmed milk in TBS-T (blocking buffer) for 1 hour, then the blocked membrane were incubated with primary antibodies overnight at 4 °C. The primary antibodies used for the western blot were as follows: rabbit antibody against RCE1 (Santa Cruz Biotechnology, Dallas, TX, USA), and rabbit antibodies against p-p38, p-38, p-JNK, p-ERK1/2 and β-actin (C Cell Signaling Technology, Danvers, USA). Afterward, the membranes were washed with TBS-T for 10 min three times and then incubated with a secondary antibody (Cell Signaling Technology, Danvers, USA) for 1 hour at room temperature. Finally, the membranes were exposed to Hyperfilm ECL (GE Healthcare) for signal detection.

### Transwell invasion assay

The transwell invasion assay was performed in Transwell chamber (BD Bioscience, San Jose, CA) according to the manufacturer’s protocol. Approximately 2 × 10^4^ cells were seeded into the top chamber of each insert and incubated at 37 °C for 48 h. Cell that invaded through the Matrigel were stained and quantified with crystal violet (Sigma-Aldrich, USA).

### Statistics

All of the data were analyzed by SPSS statistical software (version 21.0; SPSS, IBM, Armonk, NY, USA). Survival curves were plotted using the Kaplan–Meier method and analyzed by the log-rank test. The Pearson correlation test (2-tailed) was used to calculate the correlation coefficient (r) and *P* value between the RCE1 and p-p38, p-JNK, and p-ERK1/2 staining scores. Student’s *t*-test was used for comparisons. Results were considered statistically significant with *P* values <0.05.

## Results

### RCE1 expression in CRC tumor and adjacent non-tumorous tissue samples

The expression patterns of RCE1 in 244 CRC tumors and matched adjacent non-tumorous tissues were examined by IHC staining.

**Fig. 1:**
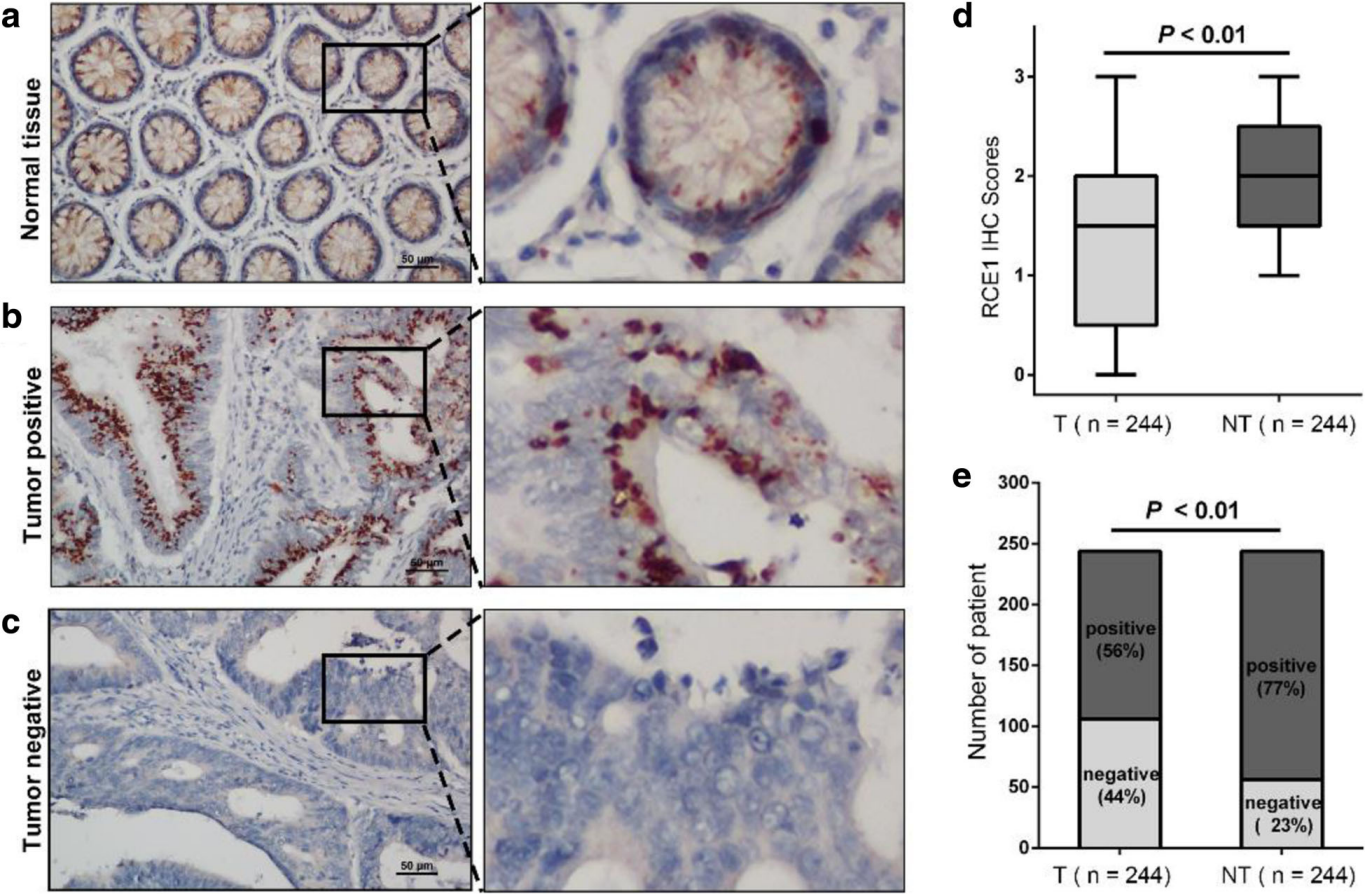
IHC analysis of RCE1 expression in CRC tumor and adjacent non-tumorous tissue samples. **a**-**c** Representative images showed strong RCE1 staining in non-tumorous tissue and tumor tissue and low RCE1 staining in tumor tissue. The *right* panels are magnified pictures (×5) of the boxed area in the *left* panels*.* Scale bars, 50 μm. **d** RCE1 expression levels were significantly higher in adjacent non-tumorous tissues (NT) compared with the CRC tissues (*p* < 0.01). **e** The positive rate of RCE1 in adjacent non-tumorous tissues was higher than that in matched CRC tissues (*p* < 0.01)

We found that different expression patterns were exhibited in the tumor and matched non-tumorous tissues. RCE1 presented a predominantly cytoplasmic pattern of immunoreactivity in the tissues (Fig. 1a - c). Our results suggested that RCE1 expression was reduced in CRC tumor tissues compared with adjacent non-tumorous tissues. Furthermore, The IHC score of RCE1 was higher in the non-tumorous tissues than that in the tumor tissues (*p* < 0.01; Fig. 1d). Moreover, RCE1 expression was high in 188 (77.0%) of the adjacent non-tumorous tissues but only in 138 (56.6%) of the tumor tissue samples (Fig. 1e). This result indicates that RCE1 expression levels were significantly decreased in the CRC tumor tissues.

### Reduction of RCE1 expression in CRC tissues predicted a poor prognosis

**Fig. 2:**
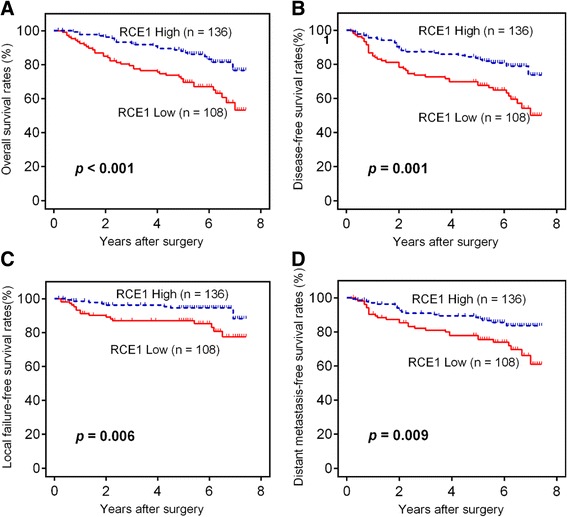
Kaplan-Meier survival estimates and log-rank tests indicated that patients with low RCE1 expression exhibited a significantly poorer prognosis. Overall survival (**a**), disease-free survival (**b**), local failure-free survival (**c**) and distant metastasis-free survival (**d**) curves, which were generated based on the RCE1 expression status in 244 CRC samples

Next, we estimated the prognostic value of RCE1 expression. We found that the overall survival (OS) of patients with low RCE1 expression was significantly shorter than that of patients with high RCE1 expression (66.7% vs 90.0%, *p* < 0.001, Fig. 2a). Similarly, the disease-free survival rates (DFS) in the patients who exhibited low RCE1 expression were significantly shorter than in the patients who exhibited high RCE1 expression (62.0% vs 85.3%, *p* = 0.001, Fig. 2b). Our study also indicated that there was a significant difference in the local failure-free survival rate (LFFS) and distant metastasis-free survival rate (DMFS) between patients with high RCE1 expression and patients with low RCE1 expression. The patients with low RCE1 expression had a higher risk of local treatment failure and distant metastasis (*p* < 0.05, Fig. 2c and d). Furthermore, univariate and multivariate Cox regression analyses were applied to verify the prognostic value of RCE1 expression. First, the RCE1 expression status and some common clinicopathological parameters were subjected to a univariate Cox regression model to determine their prognostic value. The data suggested that RCE1 expression, depth of tumor invasion, histological grade and node stage had prognostic value (Additional file 1: Table S1). These factors were then entered into a multivariate model to identify independent predictors. We found that RCE1 expression level, histological grade, depth of invasion and node stage were all independent factors of OS, and that the RCE1 expression levels, histological grade and node stage were independent prognostic factors of DFS (Table 2). Interestingly, the results were contrary to our initial expectations. The literature states that RCE1 expression is required for Ras activation. By considering the well-known carcinogenic and tumor-promoting effect of Ras activation, we initially hypothesized that RCE1 might promote CRC development by participating in Ras activation. Nevertheless, the present study conflicts with this hypothesis. Thus, we concluded that RCE1 affected CRC development but was not involved in the activation of Ras.

**Table 2:** Multivariate Analysis of overall survival (OS) and Disease-free survival (DFS) for colorectal (CRC) patients

Variables	OS		DFS	
	Hazard ratio (95% CI)	*P*-value	Hazard ratio (95% CI)	*P*-value
Depth of invasion (T3 vs T1/T2)	2.613 (1.028–3.026)	**0.044**	2.192 (0.985–4.877)	0.540
Histological grade (3 vs 1/2)	2.374 (1.260–4.472)	**0.007**	2.014 (1.102–3.679)	**0.023**
Node stage (N1/N2 vs N0)	1.793 (1.063–3.026)	**0.029**	1.859 (1.138–3.034)	**0.013**
RCE1 (high vs low)	0.511 (0.299–0.872)	**0.014**	0.572 (0.349–0.938)	**0.027**

Bold values (*p* < 0.05) are statistically significant

**Fig. 3:**
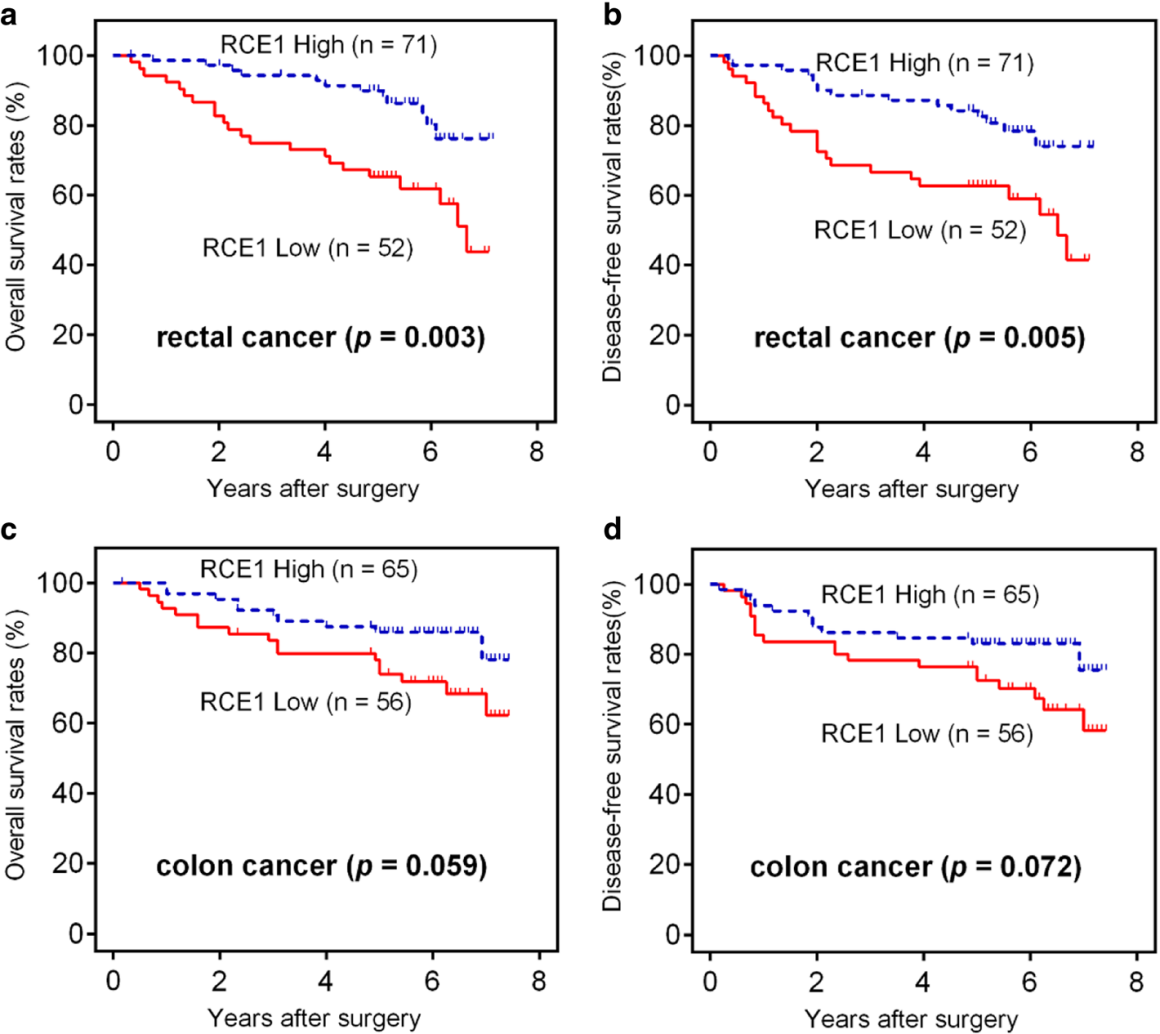
Prognostic significance assessed in rectal carcinoma and colon carcinoma by Kaplan-Meier survival estimates and log-rank tests, respectively. Comparison of the overall survival (OS) and disease-free survival (DFS) in rectal carcinoma (**a** and **b**) and colon carcinoma (**c** and **d**), respectively

Because the colon and rectum have different embryonic origins, colon cancer and rectal cancer always present different distinctive features [5, 15]. We further explored the prognostic significance of RCE1 in colon cancer and rectal cancer. Although there was a significant difference in the OS and DFS between patients who had different RCE1 expression levels in colon cancer (Fig. 3a and b) and rectal cancer (Fig. 3c and d), the RCE1 expression level had a more significant prognostic value in rectal cancer.

### Correlation of RCE1 expression with clinicopathological parameters

To understand the clinicopathological significance and potential gene function of RCE1 in CRC, we tested the correlation of the RCE1 expression status with several standard clinicopathological parameters. Our results indicated that the expression level of RCE1 was negatively correlated with the plasma levels of CEA, depth of tumor invasion, node stage and TNM stage (*p <* 0.05*,* Table 1). No significant correlation was observed between RCE1 expression and other clinicopathological parameters such as gender, age, CA19–9 expression, location and histological grade (*p* > 0.05, Table 1). High RCE1 expression levels correlated with a shallow tumor invasion depth and low node stage. Thus, RCE1 might have a role in inhibiting tumor invasion.

### Knockdown of RCE1 expression decreased p38 phosphorylation and increased the invasion capacity of CRC cells

**Fig. 4:**
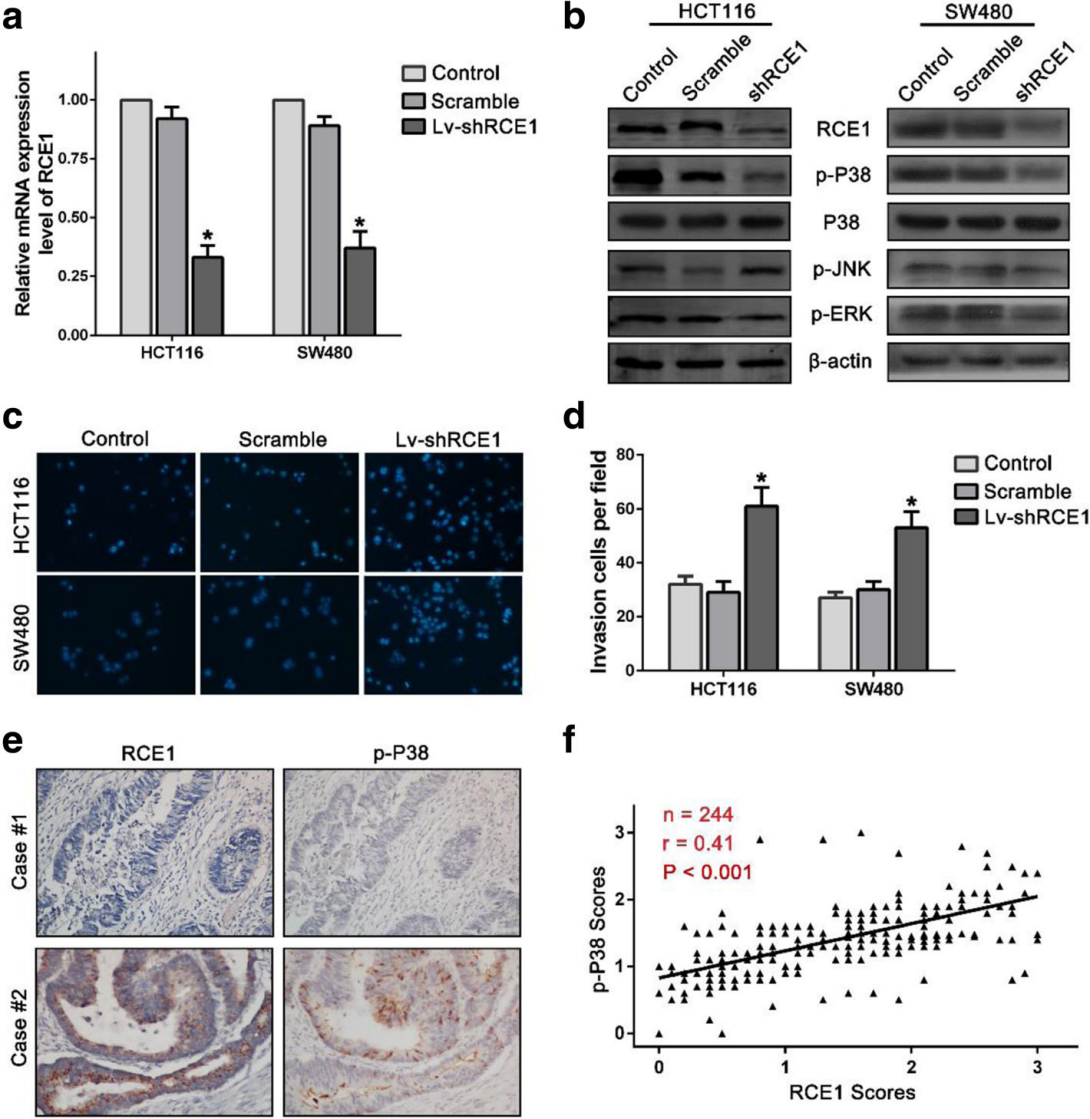
Knockdown of RCE1 increases the invasion capacity of CRC cells. **a** Real-time quantitative PCR analysis detected RCE1 expression in HCT116 and SW480 cells that were infected with an shRNA-lentivirus targeting RCE1. **b** Infection with lentivirus targeting RCE1 significantly decreased RCE1 expression and levels of phosphorylated p38. **c** and **d** Infection with lentivirus against RCE1 significantly increased the invasion capacity of HCT116 and SW480 cells, *p < 0.01. **e** Representative samples of RCE1 and p-p38 staining in the serial sections from the same tumor tissues. **f** The scatter plot shows that the RCE1 expression is positively correlated with p-p38 expression

Because existing evidence suggested that down-regulation of RCE1 expression might correlate with a reduced invasion capacity of CRC cells, we sought to verify this hypothesis. We used a shRNA lentivirus targeting RCE1 to infect and knock down RCE1 expression in HCT116 and SW480 cells (Fig. 4a and b). We then detected the invasion capacity of these cells using a transwell assay. We found that transduction of the lentivirus targeting RCE1 significantly decreased the invasiveness of HCT116 and SW480 cells (Fig. 4c and d).

Although the effect of RCE1 in CRC development might be independent of the Ras signaling pathway, we did detect the activation of members of the MAPK family, which are principally downstream of Ras signaling [16, 17], after interfering with RCE1 expression in CRC cells. We detected the phosphorylation levels of ERK1/2, JNK and p38 in RCE1-knockdown CRC cells. Our results suggested that decreased RCE1 expression reduced only p38 phosphorylation but did not affect the phosphorylation levels of either JNK or ERK1/2 (Fig. 4b). In addition, this relationship was further confirmed by IHC assays in serial sections of CRC tissues (Fig. 4e and f; Additional file 2: Figure S2 and Additional file 3: Table S2). In the serial sections, the RCE1 expression levels were positively correlated with the p38 phosphorylation levels (Fig. 4e and f) but were not correlated with the phosphorylation levels of ERK1/2 or JNK (Additional file 2: Figure S2). We also estimated the prognostic value of p-p38, p-ERK1/2 and p-JNK. We found that only the p38 phosphorylation levels were a prognosis predictor. In our study, patients with high levels of p38 phosphorylation had a better prognosis than patients with low levels of p38 phosphorylation with regard to both OS and DFS (Additional file 4: Figure 1a and b).

## Discussion

The prognosis of patients with advanced CRC who receive conventional treatment strategies remains poor. Moreover, because the traditional TNM classification system is based on the location and size of the tumor rather than on an individual basis. It is difficult to individually define a patient’s outcome. Therefore, identifying molecular prognostic predictors to determine the risks and prognoses of patients with CRC is important for guiding treatment.

Increasing evidence suggests that RCE1 is required for Ras activation [9, 10]. Abnormal activation of Ras is important in CRC development, and there have been many drugs that either have targeted the Ras signaling pathway or were affected by Ras activation [11, 18]. In the present study, we wanted to determine whether RCE1 expression could be a predictor of the prognosis of CRC patients to either guide treatment or serve as a potential target. However, the results were unexpected. Although the literature has reported that RCE1 is important for the activation of the Ras oncogene [10], our results suggested that RCE1 had an anti-tumor role in CRC. We found that the RCE1 expression levels were negatively correlated with the prognosis of CRC patients. Moreover, knockdown of RCE1 decreased the invasion capacity of CRC cells. Although it does not offer direct evidence, our finding indirectly proves that RCE1 exerted a tumor suppressor function that was independent of the Ras oncogene. Further investigation suggested that a decrease in RCE1 expression correlated with the low invasive capacity of CRC cells. RCE1 expression might affect prognosis by affecting CRC invasion. We also investigated the molecular mechanisms that were influenced by RCE1. Although RCE1 might be not correlated with Ras, we still detected the activation of Ras downstream molecules, namely, MAPK family proteins. This family is well characterized as downstream effectors of Ras. The MAPK signaling pathway has an important role in colorectal cancer development and influences apoptosis, adhesion, angiogenesis, invasion and metastasis [19]. There are three major members of the MAPK family: p38 MAP kinase (p38), the c-Jun N-terminal or stress-activated protein kinases (JNK or SAPK) and the extracellular-signal-regulated kinases (ERK) [20]. Our results showed that the expression of RCE1 was positively correlated with the phosphorylation levels of p38, but it was not correlated with the phosphorylation levels of ERK1/2 and JNK. p38 is a principle member of the MAPK family. It had been reported that p38 activation reduced the invasive ability of colon cancer cells [21]. Our data support this conclusion. IHC assays indicated that patients with low levels of phosphorylated p38 had a poorer prognosis and an increased risk of recurrence. Furthermore, we interfered with RCE1 expression in two CRC cell lines and detected a reduced invasive ability of the cells accompanied by decreased levels of phosphorylated p38. There have been some studies that reported the tumor suppressive effect of phosphorylated p38 [22, 23]. Combined with the literature, our results indirectly illustrated that a decrease in RCE1 expression might reduce the invasion capacity of CRC cells due to lower levels of phosphorylated p38.

## Conclusions

In conclusion, Our data demonstrated that RCE1 suppressed the invasive ability of CRC cells and that its expression was negatively correlated with the prognosis of CRC patients. Moreover, this correlation was more significant in rectal cancer. Furthermore, our study also indirectly indicated that RCE1 might exert a tumor suppressing function as a result of increasing levels of phosphorylated p38. Despite the lack of direct evidence, our study also provided clues about the function of the RCE1-p38 signaling pathway in colorectal cancer.

## Additional files

Additional file 1: Table S1. Univariate Analysis of overall survival (OS) and Disease-free survival (DFS) for colorectal (CRC) patients. (DOCX 15 kb)

Additional file 2: Figure S2. RCE1 expression did not correlate with the phosphorylation of JNK and ERK1/2 in 100 CRC tissues. (A and C) Serial sections of human CRC tissues were subjected to immunohistochemistry (IHC) staining with antibodies against RCE1, P-JNK and P-Erk1/2. (B and D) Scatter plots indicated that RCE1 expression did not correlate with the phosphorylation level of JNK and ERK1/2. (DOCX 13 kb)

Additional file 3: Table S2. Correlation of RCE1 expression with Phospho-MAPK Family in CRC tissue specimens. (DOCX 111 kb)

Additional file 4: Figure S1. Kaplan-Meier survival analysis and log-rank test indicated that the survival of patients with high phosphorylation levels of p38 was significantly better than that of patients with low phosphorylation levels. OS (A) and DFS (B) curves were generated based on the P-p38 phosphorylation statuses of 244 CRC samples. (DOCX 1418 kb)

## Abbreviations

CRC: Colorectal carcinoma
DFS: Disease-free survival
IHC: Immunohistochemistry
LFFS: Local failure-free survival
MFS: Metastasis-free survival
OS: Overall survival
RCE1: Ras-converting enzyme 1

## References

[1] Jemal A, Bray F, Center MM, Ferlay J, Ward E, Forman D (2011). Global cancer statistics. CA Cancer J Clin.

[2] Ross JS, Torres-Mora J, Wagle N, Jennings TA, Jones DM (2010). Biomarker-based prediction of response to therapy for colorectal cancer: current perspective. Am J Clin Pathol.

[3] Sanz-Garcia E, Sauri T, Tabernero J, Macarulla T (2015). Pharmacokinetic and pharmacodynamic evaluation of aflibercept for the treatment of colorectal cancer. Expert Opin Drug Metab Toxicol.

[4] Brenner H, Kloor M, Pox CP (2014). Colorectal cancer. Lancet.

[5] Tamas K, Walenkamp AM, de Vries EG, van Vugt MA, Beets-Tan RG, van Etten B, de Groot DJ, Hospers GA. Rectal and colon cancer: Not just a different anatomic site. Cancer treatment reviews. 2015;41(8):671–79. 10.1016/j.ctrv.2015.06.007 26145760

[6] Manolaridis I, Kulkarni K, Dodd RB, Ogasawara S, Zhang Z, Bineva G, O'Reilly N, Hanrahan SJ, Thompson AJ, Cronin N, Iwata S, Barford D (2013). Mechanism of farnesylated CAAX protein processing by the intramembrane protease Rce1. Nature.

[7] Prior IA, Hancock JF (2012). Ras trafficking, localization and compartmentalized signalling. Semin Cell Dev Biol.

[8] Bergo MO, Ambroziak P, Gregory C, George A, Otto JC, Kim E, Nagase H, Casey PJ, Balmain A, Young SG (2002). Absence of the CAAX endoprotease Rce1: effects on cell growth and transformation. Mol Cell Biol.

[9] Burrows JF, Kelvin AA, McFarlane C, Burden RE, McGrattan MJ, De la Vega M, Govender U, Quinn DJ, Dib K, Gadina M, Scott CJ, Johnston JA (2009). USP17 regulates Ras activation and cell proliferation by blocking RCE1 activity. J Biol Chem.

[10] Jaworski J, Govender U, McFarlane C, de la Vega M, Greene MK, Rawlings ND, Johnston JA, Scott CJ, Burrows JF (2014). A novel RCE1 isoform is required for H-Ras plasma membrane localization and is regulated by USP17. Biochem J.

[11] Douillard JY, Oliner KS, Siena S, Tabernero J, Burkes R, Barugel M, Humblet Y, Bodoky G, Cunningham D, Jassem J, Rivera F, Kocakova I, Ruff P, Blasinska-Morawiec M, Smakal M, Canon JL, Rother M, Williams R, Rong A, Wiezorek J, Sidhu R, Patterson SD (2013). Panitumumab-FOLFOX4 treatment and RAS mutations in colorectal cancer. N Engl J med.

[12] He L, Zhou X, Qu C, Hu L, Tang Y, Zhang Q, Liang M, Hong J (2014). Musashi2 predicts poor prognosis and invasion in hepatocellular carcinoma by driving epithelial-mesenchymal transition. J Cell Mol med.

[13] Jiang P, Tang Y, He L, Tang H, Liang M, Mai C, Hu L, Hong J (2014). Aberrant expression of nuclear KPNA2 is correlated with early recurrence and poor prognosis in patients with small hepatocellular carcinoma after hepatectomy. Med Oncol.

[14] Hong J, Hu K, Yuan Y, Sang Y, Bu Q, Chen G, Yang L, Li B, Huang P, Chen D, Liang Y, Zhang R, Pan J, Zeng YX, Kang T (2012). CHK1 targets spleen tyrosine kinase (L) for proteolysis in hepatocellular carcinoma. J Clin Invest.

[15] Fazeli MS, Keramati MR (2015). Rectal cancer: a review. Med J Islam Repub Iran.

[16] Mor A, Philips MR (2006). Compartmentalized Ras/MAPK signaling. Annu rev Immunol.

[17] Roberts MJ, Troutman JM, Chehade KA, Cha HC, Kao JP, Huang X, Zhan CG, Peterson YK, Subramanian T, Kamalakkannan S, Andres DA, Spielmann HP (2006). Hydrophilic anilinogeranyl diphosphate prenyl analogues are Ras function inhibitors. Biochemistry.

[18] Zhang C, Spevak W, Zhang Y, Burton EA, Ma Y, Habets G, Zhang J, Lin J, Ewing T, Matusow B, Tsang G, Marimuthu A, Cho H, Wu G, Wang W, Fong D, Nguyen H, Shi S, Womack P, Nespi M, Shellooe R, Carias H, Powell B, Light E, Sanftner L, Walters J, Tsai J, West BL, Visor G, Rezaei H, Lin PS, Nolop K, Ibrahim PN, Hirth P, Bollag G (2015). RAF inhibitors that evade paradoxical MAPK pathway activation. Nature.

[19] Fang JY, Richardson BC (2005). The MAPK signalling pathways and colorectal cancer. Lancet Oncol.

[20] Derbal Y (2014). State machine modeling of MAPK signaling pathways. Conf Proc IEEE Eng med Biol Soc.

[21] Urosevic J, Garcia-Albeniz X, Planet E, Real S, Cespedes MV, Guiu M, Fernandez E, Bellmunt A, Gawrzak S, Pavlovic M, Mangues R, Dolado I, Barriga FM, Nadal C, Kemeny N, Batlle E, Nebreda AR, Gomis RR (2014). Colon Cancer cells colonize the lung from established liver metastases through p38 MAPK signalling and PTHLH. Nat Cell Biol.

[22] Lu WJ, Chua MS, So SK. Suppression of ATAD2 inhibits hepatocellular carcinoma progression through activation of p53- and p38-mediated apoptotic signaling. Oncotarget. 2015;6(39):41722–35. 10.18632/oncotarget.6152 PMC4747184 26497681

[23] Sun P, Yoshizuka N, New L, Moser BA, Li Y, Liao R, Xie C, Chen J, Deng Q, Yamout M, Dong MQ, Frangou CG, Yates JR, Wright PE, Han J (2007). PRAK is essential for ras-induced senescence and tumor suppression. Cell.

