# Supplementary material for: Reduced RCE1 expression predicts poor prognosis of colorectal carcinoma
Source: BMC Cancer. 2017 Jun 14;17:414. doi: 10.1186/s12885-017-3393-3 (PMC5471898; doi:10.1186/s12885-017-3393-3)
Supplement: Supplementary file 1 — Univariate Analysis of overall survival (OS) and Disease-free survival (DFS) for colorectal (CRC) patients. (DOCX 15 kb) [file 12885_2017_3393_MOESM1_ESM.docx]

**Table S1** Univariate Analysis of overall survival（OS）and Disease-free survival (DFS) for colorectal (CRC) patients.

| Variables | OS | |  | DFS | |
| --- | --- | --- | --- | --- | --- |
|  | Hazard ratio (95% CI) | *P*-value |  | Hazard ratio (95% CI) | *P*-value |
| Gender (male vs. female) | 1.210 (0.713-2.054) | 0.479 |  | 1.251 (0.763-2.051) | 0.375 |
| Age (y) (> 60 vs. ≤ 60 ) | 1.387 (0.839-2.293) | 0.202 |  | 1.015 (0.632-1.630) | 0.951 |
| CEA (ng/ml) (> 5 vs. ≤ 5) | 1.694 (0.990-2.747) | 0.055 |  | 1.797 (1.114-2.900) | **0.016** |
| CA19-9 (U/ml) (> 37 vs. ≤ 37) | 1.224 (0.699-2.142) | 0.480 |  | 1.497 (0.903-2.480) | 0.118 |
| Location (colon vs. rectum) | 1.389 (0.835-2.311) | 0.206 |  | 1.415 (0.879-20278) | 0.153 |
| Depth of invasion (T3 vs. T1/T2) | 3.647 (1.461-9.107) | **0.006** |  | 3.014 (1.380-6.582) | **0.006** |
| Histological grade (III vs. I/II) | 2.821 (1.507-5.283) | **0.001** |  | 2.413 (1.330-4.464) | **0.005** |
| Node stage (N1/N2 vs. N0) | 2.419 (1.457-4.017) | **0.001** |  | 2.689 (1.618-4.378) | **0.004** |
| RCE1 (high vs low) | 0.407 (0.241-0.687) | **0.001** |  | 0.457 (0.282-0.740) | **0.001** |
| P-p38 (high vs low) | 0.474 (0.278-0.810) | **0.006** |  | 0.525 (0.322-0.856) | **0.010** |

CRC colorectal cancer, HR hazard ratio, CI confidence interval. Bold values **(p <0.05)** are statistically significant
