# Supplementary material for: Reduced RCE1 expression predicts poor prognosis of colorectal carcinoma
Source: BMC Cancer. 2017 Jun 14;17:414. doi: 10.1186/s12885-017-3393-3 (PMC5471898; doi:10.1186/s12885-017-3393-3)
Supplement: Supplementary file 2 — RCE1 expression did not correlate with the phosphorylation of JNK and ERK1/2 in 100 CRC tissues. (A and C) Serial sections of human CRC tissues were subjected to immunohistochemistry (IHC) staining with antibodies against RCE1, P-JNK and P-Erk1/2. (B and D) Scatter plots indicated that RCE1 expression did not correlate with the phosphorylation level of JNK and ERK1/2. (DOCX 13 kb) [file 12885_2017_3393_MOESM2_ESM.docx]

**Figure S2**


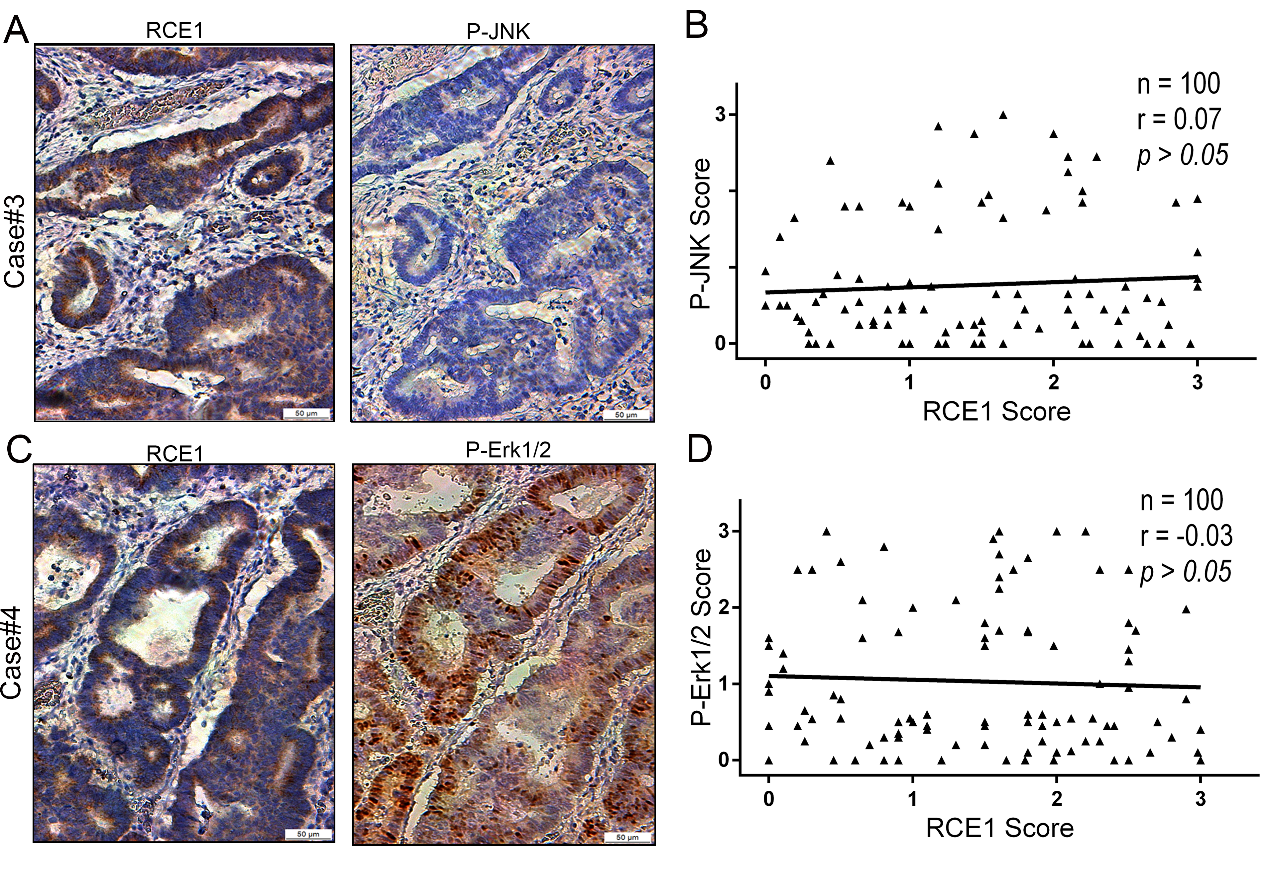


**Supplementary Fig. 2 RCE1 expression did not correlate with the phosphorylation of JNK and ERK1/2 in 100 CRC tissues**. **(A and C)** Serial sections of human CRC tissues were subjected to immunohistochemistry (IHC) staining with antibodies against RCE1, P-JNK and P-Erk1/2. **(B and D)** Scatter plots indicated that RCE1 expression did not correlate with the phosphorylation level of JNK and ERK1/2.
