# Supplementary material for: Reduced RCE1 expression predicts poor prognosis of colorectal carcinoma
Source: BMC Cancer. 2017 Jun 14;17:414. doi: 10.1186/s12885-017-3393-3 (PMC5471898; doi:10.1186/s12885-017-3393-3)
Supplement: Supplementary file 3 — Correlation of RCE1 expression with Phospho-MAPK Family in CRC tissue specimens. (DOCX 111 kb) [file 12885_2017_3393_MOESM3_ESM.docx]

**Table S2** Correlation of RCE1 expression with Phospho-MAPK Family in CRC tissue specimens

|  | P-P38 | | P-JNK | | P-ERK | |
| --- | --- | --- | --- | --- | --- | --- |
|  | **Low** | **High** | **Low** | **High** | **Low** | **High** |
| RCE1 |  |  |  |  |  |  |
| Low (%) | 74 (68.5%) | 34 (31.5%) | 38 (78.1) | 9 (21.9) | 31 (73.8%) | 11 (26.2%) |
| High (%) | 49 (36.0%) | 87 (64.0%) | 38 (66.7) | 15 (33.3) | 38 (64.9%) | 20 (35.1%) |
| *P*-value | **<0.001** | | 0.351 | | 0.511 | |

Bold values **(*p* < 0.05)** are statistically significant.
