# Supplementary material for: Reduced RCE1 expression predicts poor prognosis of colorectal carcinoma
Source: BMC Cancer. 2017 Jun 14;17:414. doi: 10.1186/s12885-017-3393-3 (PMC5471898; doi:10.1186/s12885-017-3393-3)
Supplement: Supplementary file 4 — Kaplan-Meier survival analysis and log-rank test indicated that the survival of patients with high phosphorylation levels of p38 was significantly better than that of patients with low phosphorylation levels. OS (A) and DFS (B) curves were generated based on the P-p38 phosphorylation statuses of 244 CRC samples. (DOCX 1418 kb) [file 12885_2017_3393_MOESM4_ESM.docx]

**Figure S1**


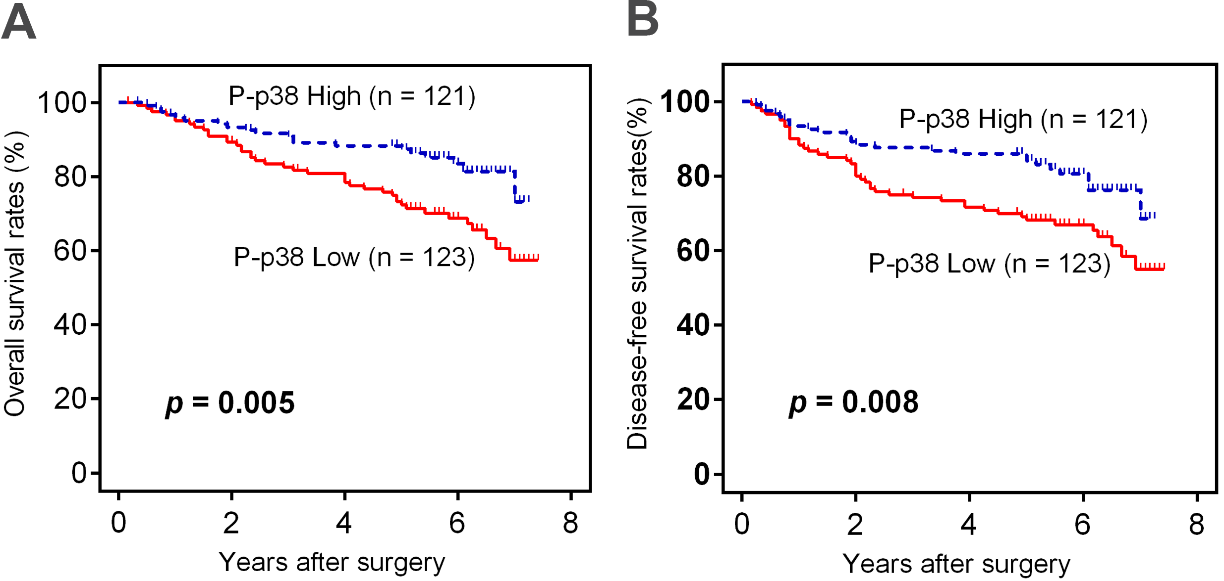


**Supplementary Fig. 1 Kaplan-Meier survival analysis and log-rank test indicated that the survival of patients with high phosphorylation levels of p38 was significantly better than that of patients with low phosphorylation levels.** OS **(A)** and DFS **(B)** curves were generated based on the P-p38 phosphorylation statuses of 244 CRC samples.
